# Magnitude and persistence of higher estrus-associated temperatures in beef heifers and suckled cows

**DOI:** 10.1093/jas/skae079

**Published:** 2024-03-19

**Authors:** Megan D Mills, Abigayle B Pollock, Ian E Batey, Michael A O’Neil, F Neal Schrick, Rebecca R Payton, Sarah E Moorey, Pablo Fioravanti, Will Hipsher, Saulo M Zoca, J Lannett Edwards

**Affiliations:** Department of Animal Science, The University of Tennessee Institute of Agriculture and AgResearch, Knoxville, TN 37996, USA; Department of Animal Science, The University of Tennessee Institute of Agriculture and AgResearch, Knoxville, TN 37996, USA; Department of Animal Science, The University of Tennessee Institute of Agriculture and AgResearch, Knoxville, TN 37996, USA; Office of Innovative Technologies—Research Computing Support, The University of Tennessee, Knoxville, TN 37996, USA; Department of Animal Science, The University of Tennessee Institute of Agriculture and AgResearch, Knoxville, TN 37996, USA; Department of Animal Science, The University of Tennessee Institute of Agriculture and AgResearch, Knoxville, TN 37996, USA; Department of Animal Science, The University of Tennessee Institute of Agriculture and AgResearch, Knoxville, TN 37996, USA; Department of Animal Science, The University of Tennessee Institute of Agriculture and AgResearch, Knoxville, TN 37996, USA; Department of Animal Science, The University of Tennessee Institute of Agriculture and AgResearch, Knoxville, TN 37996, USA; Department of Animal Science, The University of Tennessee Institute of Agriculture and AgResearch, Knoxville, TN 37996, USA; Department of Animal Science, The University of Tennessee Institute of Agriculture and AgResearch, Knoxville, TN 37996, USA

**Keywords:** baseline temperature, elevated vaginal temperature, estrus, heat, heifers, higher estrus-associated temperatures, suckled beef cows

## Abstract

Higher estrus-associated temperatures (**HEAT**) are a hallmark feature in sexually active females. The overarching aim of this study was to characterize the variability, magnitude, and persistence of HEAT in heifers and suckled beef cows as well as identify associated factors when occurring during thermoneutral conditions at the onset of the spring breeding season. In both heifers and cows, estrus was induced using a 7-d controlled internal drug release (**CIDR**)-PGF_2α_ protocol. Vaginal temperature after prostaglandin F_2α_ administration was recorded every 5 min using a Thermochron iButton affixed to a blank CIDR (containing no progesterone). Estrus was defined as when a heifer first stood to be mounted or when a cow had an Estrotect patch score of 3 or 4. Level of HEAT varied among individual animals. When comparing common HEAT variables using a mixed model with date nested within a year, maximum HEAT (39.9 ± 0.1 and 40.0 ± 0.1 °C) and duration (15.5 ± 0.8 and 15.4 ± 0.7) were similar in heifers and cows, respectively. However, the magnitude and persistence of HEAT differed. Total area under the HEAT curve was 117.1 ± 13.5 and 158.7 ± 12.3 for heifers vs cows, respectively (*P *= 0.0571). Further, 42.9% of heifers and 49% of cows had maximum HEAT ≥ 40 °C which persisted up to 6.5 and 10 h, respectively. When ambient conditions were predominantly thermoneutral, temperature humidity index had minimal impact on HEAT (mixed model, repeated measures over time). Toward identifying associated factors with different aspects of HEAT using best fit hierarchical linear regression models, baseline vaginal temperature and baseline duration were the most highly associated independent variables. Follicle size, estradiol and progesterone levels, and other available animal-related variables (e.g., age, weight, hair coat score) explained only a small amount of variation in HEAT. In summary, level of HEAT varies in estrus females even under thermoneutral conditions. Because HEAT can persist for an extended time, direct effects on fertility important components are unavoidable. Whether HEAT is a good or bad component of the periovulatory microenvironment is the basis of ongoing and future studies.

## Teaser

Sexually active cattle: Hotter for longer than one may think.

## Introduction

When striving for a pregnancy, estrus is a critically important event, even when occurring before fixed-time artificial insemination (**FTAI**) (Whittier et al., 2013; Colazo et al., 2018; Oliveira Filho et al., 2020). A threshold level of estradiol, achieved soon after the female first stands to be mounted, triggers the gonadotropin-releasing hormone (**GnRH**)-induced luteinizing hormone (**LH**) surge through positive endocrine feedback (Short et al., 1979). The oocyte contained within the mature Graafian follicle resumes meiosis and progresses to metaphase II (Kruip et al., 1983). The LH surge also marks the beginnings of corpus luteum (**CL**) formation and initiates processes important for ovulation ~30 h thereafter (Smith et al., 1994; Giordano et al., 2013).

While the most definitive sign of estrus is the willingness of a female to stand to be mounted by herd mates or a bull (Foote, 1975; At-Taras and Spahr, 2001; Roelofs et al., 2010), estrus females are quite active, walking up to four times more than non-estrual herd mates (Kiddy, 1977; Lewis and Newman, 1984; Redden et al., 1993; Diskin and Sreenan, 2000). Interestingly, both mounting (Rorie et al., 2002) and walking activity have been related to higher pregnancy outcomes in cattle (López-Gatius et al., 2005; Burnett et al., 2018; Madureira et al., 2019).

Higher estrus-associated temperatures (**HEAT**) are a hallmark feature of estrus-active females (Wrenn et al., 1958; Kyle et al., 1998; Wang et al., 2020). In some instances, HEAT may approach or exceed 40 °C (Cooper-Prado et al., 2011; Talukder et al., 2014; Kim et al., 2023). During times of moderate to severe heat stress, HEAT has been associated with negative fertility outcomes (Putney et al., 1989; De Rensis et al., 2002). However, under conditions closer to thermoneutral, the level of HEAT has been associated with positive fertility outcomes. In cows that exhibited estrus, Fallon (1962) reported greater pregnancy outcomes in cows that had rectal temperatures ranging from 38.7 to 40.5 °C immediately before AI compared to those with rectal temperatures ranging from 37.1 to 38.6 °C (73.5% vs. 60.2%, respectively). Furthermore, rectal temperature at FTAI has also been positively associated with pregnancy outcome. Per each unit increase in rectal temperature, pregnancy odds increased by 1.9 and 1.5 in different populations of *Bos indicus* and *Bos taurus* cows, respectively (Liles et al., 2022). Notably, the highest pregnancy outcomes occurred in cows with rectal temperatures exceeding 40 °C immediately before insemination.

While the occurrence of HEAT is nondisputed, the literature remains scant on studies evaluating the extent to which HEAT, occurring during thermoneutral conditions, may be functionally impactful on pregnancy outcomes. Toward filling this critical knowledge gap, further efforts that are described herein in beef heifers and suckled beef cows were put forth to 1) examine the extent to which HEAT varies among individual animals (most studies report an average temperature or average temperature change), 2) better define the magnitude and persistence of HEAT in estrual females (i.e., amount of time ≥39.5, ≥40.0, and ≥41.0 °C), and 3) identify HEAT-associated factors using available animal and environmental data. The extent to which HEAT is comparable in heifers versus suckled beef cows at the onset of a spring breeding season, where conditions are mostly thermoneutral and pregnancy outcomes of 50% or greater are expected, was also evaluated.

## Materials and Methods

Institutional animal care and use approval at the University of Tennessee, Knoxville, was obtained before the onset of studies described below.

### Study one: HEAT in beef heifers

#### Animals and synchronization protocol

**Figure 1.**
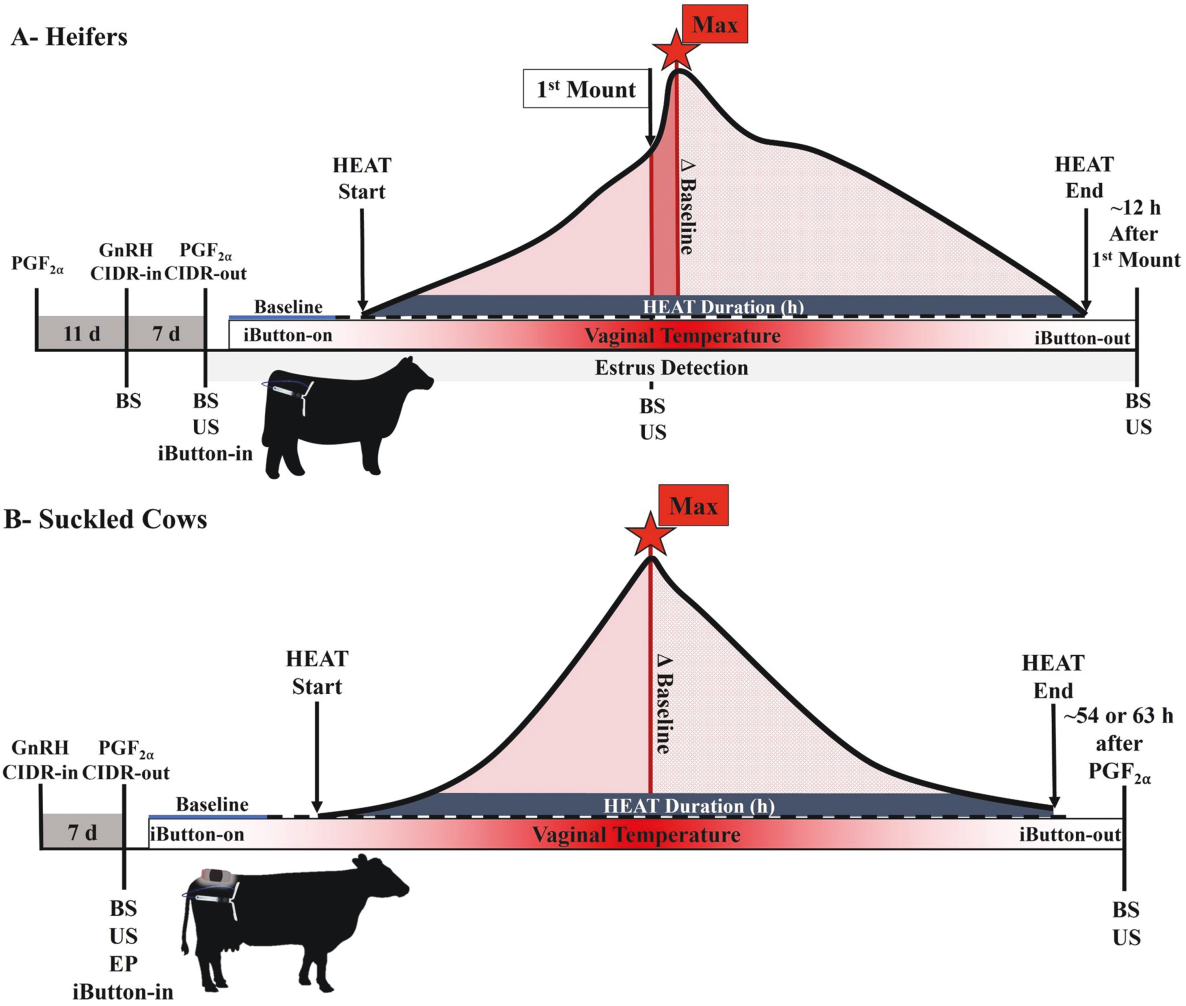
Schematic depicting synchronization protocol, blood sample collections, ovarian ultrasound evaluations, and timing related to when the vaginal temperature was recorded every 5 min after prostaglandin F__2_⍺_ (PGF__2_⍺_) administration relative to HEAT curve in beef heifers (panel A) and suckled cows (panel B). Representative HEAT curve is one that was derived after averaging each animal’s complete HEAT curve from the 11 h prior to maximum HEAT and up to 11 h after maximum HEAT. GnRH: gonadotropin-releasing hormone; CIDR: controlled internal drug release; BS: blood sample; US: ovarian ultrasound; EP: Estrotect patch.

Virgin Angus heifers across two different years (year 1, *n* = 38; year 2, *n* = 28; total *n* = 66) during the month of April and located at a University of Tennessee AgResearch and Education Center were grazed on mixed grass pastures and had ad libitum access to hay. Average heifer age was 1.2 ± 0.1 yr. Estrus was synchronized according to Figure 1A and defined as the time when a heifer first stood to be mounted by another. Depending on the year, prostaglandin F_2α_ (**PGF**_**2α**_) administered was dinoprost tromethamine (25 mg i.m.; Lutalyse, Zoetis, Parsippany, NJ, USA) or cloprostenol (500 μg i.m., Estrumate, Merck, Rahway, NJ, USA). Eleven days later, gonadotropin-releasing hormone (**GnRH**) was administered (100 μg i.m., gonadorelin hydrochloride, Factrel, Zoetis or 100 μg i.m., gonadorelin diacetate tetrahydrate, Cystorelin, Boehringer Ingelheim, Duluth, GA, USA). A controlled internal drug release (**CIDR**) device was placed intravaginally (1.38 g progesterone, Eazi-Breed CIDR, Zoetis). Seven days later, the CIDR was removed, PGF_2α_ was administered, and an iButton that had been previously affixed to a blank CIDR (containing no progesterone) was placed intravaginally. Beginning ~24 h after PGF_2α_, estrus activity was visually assessed hourly until the first heifer displayed signs of estrus at which point monitoring was continuous by a team of individuals on a rotating schedule.

#### Ovarian ultrasound

The largest diameter of follicles > 5 mm was recorded using an IBEX EVO II ultrasound and eL7 linear probe (E.I Medical Imaging, Loveland, CO, USA) at the time of PGF_2α_ administration, first standing mount, and 11.9 ± 0.1 h after first standing mount.

#### Vaginal and ambient temperature data

The vaginal temperature was recorded using a Thermochron iButton 1922L data logger (Embedded Data Systems, Lawrenceburg, KY, USA) affixed to a blank CIDR using heat shrink tubing (Burdick et al., 2012). Beyond Burdick, paraffin wax was placed beneath where iButton was to be positioned to create a wax seal when heat was applied to shrink tubing. Paraffin wax was also inserted in the open spaces, on top and bottom of shrink tubing, where tubing contacted the blank CIDR. Hot glue was then placed along the top and bottom edge of the shrink tubing to create a final seal. The vaginal temperature was recorded every 5 min (0.065 °C resolution) beginning 4 to 12 h after iButton insertion and continued until iButton removal (11.9 ± 0.1 h after heifer was first observed to stand to be mounted by another). No iButton failures occurred. Onsite ambient temperature and humidity were recorded hourly using the HOBO U23 Pro v2 data logger (Onset Computer Corporation, Bourne, MA, USA) from PGF_2α_ administration to the time that the last iButton was removed. Temperature humidity index (**THI**) was calculated per Abbott et al. (2018).

#### Baseline and higher estrus-associated temperature variables of interest

A baseline temperature was calculated for each heifer by averaging vaginal temperature beginning 4 to 12 h after PGF_2α_ administration and continuing up to the next 24 h of recorded data. Start of HEAT was noted when vaginal temperature increased 0.1 °C above baseline and continued to increase thereafter. End of HEAT was defined as the time when vaginal temperature returned to baseline or when iButton was removed. Other higher estrus-associated temperature variables taken at a singular point in time (i.e., the first mount or maximum HEAT vaginal temperature), or when expressed as a change from baseline, or as a change from another timepoint are defined in Table 1. Duration-related HEAT variables in Table 1 describe the length of time (h) between two HEAT-related events. Rate of temperature change was calculated using a change in vaginal temperature (**VTp**) divided by the change in time, ΔVTp/Δt (Wurster and McCook, 1969). Area under the curve (**AUC**) was calculated for different portions of the HEAT-related increase according to Pruessner et al. (2003) using the trapezoid formula with respect to the increase (**AUC1**) where baseline temperature was the reference point to calculate changes over time. In total, 49 of the 66 heifers stood to be mounted by another (74% estrus expression). HEAT-related variables from the first mount to HEAT end were available for all 49 estrual heifers. An unexpected event elevated the body temperature of 11 estrual heifers in the hours before they first stood to be mounted. Thus, any HEAT variables involving HEAT start were limited to 38 heifers.

**Table 1.** HEAT and other variables of interest in beef heifers

HEAT variables	*n* ^1^	Definition	Range	Mean	SEM	SD
Vaginal temperature (°C)						
First mount VTp^2^	49	Vaginal temperature at first observed mount	38.2 to 40.7	39.4	0.1	0.5
Baseline change	49	First mount VTp minus baseline VTp	−0.1 to 2.0	0.9	0.1	0.5
Maximum HEAT	49	Maximum HEAT VTp	39.0 to 41.1	39.9	0.1	0.5
Baseline change	49	Maximum HEAT minus baseline VTp	0.4 to 2.6	1.4	0.1	0.5
First mount to maximum HEAT change in VTp	49	Maximum HEAT minus the first Mount VTp	0.0 to 1.7	0.6	0.05	0.3
Duration (h)						
HEAT start^3^ to the first mount	38	HEAT start to the first mount	−0.1^4^ to 15.9	5.9	0.6	3.9
HEAT start to max	38	HEAT start to Maximum HEAT	0.3 to 16.3	7.0	0.7	4.0
First mount to max	49	First mount to maximum HEAT	−7.8^5^ to 12.1	1.2	0.5	3.2
First mount to HEAT end	49	First mount to HEAT end^6^	1.2 to 13.3	9.6	0.4	3.1
Max to HEAT end	49	Maximum HEAT to HEAT end	0.2 to 12.2	8.5	0.5	3.4
HEAT length	38	HEAT start to HEAT end	3.9 to 27.5	15.5	0.7	4.1
Rate of temperature change (°C/h)^7^						
Rate of change 1	37	HEAT start to the first mount	−0.04 to 1.7	0.2	0.04	0.3
Rate of change 2	38	HEAT start to maximum HEAT	0.1 to 3.1	0.3	0.1	0.5
Rate of change 3	48	First mount to maximum HEAT	−0.04 to 3.1	1.1	0.1	0.8
Rate of change 4	49	Maximum HEAT to HEAT end	−0.5 to 0.1	−0.2	0.02	0.1
Area under the curve (AUC)						
AUC 1	37	HEAT start to the first mount	0.0 to 105.7	37.1	4.9	29.7
AUC 2	38	HEAT start to maximum HEAT	2.3 to 129.7	48.6	5.2	32.0
AUC 3	49	First mount to HEAT end	3.6 to 202.0	88.7	6.9	48.1
AUC 4	49	Maximum HEAT to HEAT end	1.2 to 171.8	75.1	6.0	42.3
AUC 5	38	HEAT start to HEAT end	11.9 to 218.0	117.9	7.8	48.1
Other						
Baseline VTp	49	Average of up to 24 h of recorded data beginning 4 to 12 h after PGF_2α_ administration	38.1 to 38.9	38.5	0.02	0.2
Baseline duration	38	PGF_2α_ to HEAT start	27.5 to 76.4	49.7	2.1	13.1

^1^Number of observations.

^2^Vaginal temperature.

^3^HEAT start was defined as the first time point when vaginal temperature was 0.1 °C above baseline.

^4^One heifer stood to be mounted approximately 6 min prior to HEAT start.

^5^Two heifers reached maximum HEAT before standing to be mounted.

^6^HEAT end was defined as the time when a heifer returned to baseline after first standing mount or when the iButton was removed.

^7^Rate of change defined as the change in vaginal temperature divided by the change in time.

### Study two: HEAT in suckled beef cows

#### Animals and synchronization protocol

Study outcomes are derived from a subset of Angus and Angus dominant crossbred cows that underwent synchronization as a part of a larger study where treatments were applied immediately before FTAI. Thus, opportunity to evaluate HEAT was limited to ~63.4 ± 0.6 h (first year) and 53.6 ± 1.0 h (second year) after PGF_2α_ administration. Cows were maintained at three University of Tennessee AgResearch and Education Centers: location 1 (*n* = 120; 35.84°N, −85.07 °W), location 2 (*n* = 120; 35.71°N, −86.9507°W), location 3 (*n* = 80; 36.28°N, −86.50°W) on mixed grass pastures. Across the 2 yr (month of April and April until first part of May, first and second years, respectively), 320 primiparous and multiparous beef cattle, ranging in age from 2 to 14 (4.9 ± 2.4) yr, body weight from 401.9 to 907.2 (604.5 ± 95.0) kg, and days postpartum from 34 to 119 (76.8 ± 19.2) d were utilized. The estrus synchronization scheme is depicted in Figure 1B. Gonadotropin-releasing hormone was administered (100 µg i.m., Cystorelin, Boehringer Ingelheim) and a CIDR was placed intravaginally (1.38 g progesterone; Eazi-Breed CIDR; Zoetis). Seven days later, the CIDR was removed, and PGF_2α_ (25 mg i.m., Lutalyse HighCon (Zoetis) or 500 mcg SynchSure (Boehringer Ingelheim) was administered. At this time, an iButton affixed to a blank CIDR as described above was placed intravaginally, and an Estrotect patch (**EP**) was placed on the tailhead of each cow (Estrotect; Rockway Inc; Spring Valley, WI, USA).

#### Body condition score, hair coat score, and ovarian ultrasound

Body condition score was assigned according to Richards et al. (1986). A hair coat score was recorded according to the American Angus Association hair shedding scoring guide. Ovarian ultrasound was conducted as described above and performed at PGF_2α_ administration and 63.4 ± 0.6 h (first year) or 53.6 ± 1.0 h (second year) after PGF_2α_ administration.

#### Vaginal and ambient temperature data

Cow vaginal temperature was recorded every 5 min (0.065 °C resolution) beginning 7 to 33 h after iButton insertion and continued until 63.4 ± 0.6 h (first year) or 53.6 ± 1.0 h (second year) after PGF_2α_ administration when iButtons were removed. In year 1, no iButton failures occurred; in year 2, two iButtons failed to record data (318 out of 320; 99% recording success). Ambient temperature and humidity data were collected from PGF_2α_ administration to GnRH administration, and THI was calculated consistent with study one.

#### Expression of estrus

EPs, after removal occurring at 63.4 ± 0.6 h (first year) or 53.6 ± 1.0 h (second year) after PGF_2α_ administration, were visually scored for mounting activity consistent with Franco et al. (2018). Cows having EP scores of 3 or 4 (>50 to 75% or more of patch surface rubbed off) were defined as estrual (*n* = 137/318, 43.1% overall) as were cows that were missing an EP but were visually observed to stand for mounting (*n* = 6).

#### Baseline and higher estrus-associated temperature variables of interest

Baseline temperature was calculated for each cow by averaging vaginal temperature beginning 7 to 33 h after PGF_2α_ administration and continuing up to the next 20 h of recorded data. Of the 143 estrual cows, 4 cows did not have sufficient baseline data and 8 cows did not exhibit HEAT. Start of HEAT was defined as time when vaginal temperature increased 0.1 °C above baseline and continued thereafter. End of HEAT was defined as time when vaginal temperature returned to baseline. Rate of vaginal temperature change and different areas under the HEAT curve (**AUC**) were calculated as described previously for heifers. Higher estrus-associated temperature variables of interest where data were available from HEAT start to maximum HEAT (*n* = 100) or HEAT start to HEAT end (*n* = 46) are defined in Table 2. There were 31 other cows where HEAT started to increase in the few hours before iButton removal but had not reached maximum HEAT. Data from this subset of estrual cows were used to calculate baseline vaginal temperature and duration.

**Table 2.** HEAT and other variables of interest in suckled beef cows

HEAT variables	*n* ^1^	Definition	Range	Mean	SEM	SD
Vaginal temperature (°C)						
Maximum HEAT	100	Maximum HEAT VTp^2^	39.1 to 41.4	40.0	0.05	0.5
Baseline change	100	Maximum HEAT minus baseline VTp	0.4 to 2.8	1.5	0.05	0.5
Duration (h)						
HEAT start^3^ to max	100	HEAT start to maximum HEAT	0.7 to 23.9	7.2	0.4	4.4
Max to HEAT end	46	Maximum HEAT to HEAT end^4^	2.5 to 17.6	8.1	0.5	3.4
HEAT length	46	HEAT start to HEAT end	6.2 to 26.2	15.5	0.6	4.3
Rate of temperature change (°C/h)^5^						
Rate of change 1	100	HEAT start to maximum HEAT	0.04 to 1.6	0.3	0.02	0.2
Rate of change 2	46	Maximum HEAT to HEAT end	−0.6 to −0.05	−0.2	0.02	0.1
Area under the curve (AUC)						
AUC 1	100	HEAT start to Maximum HEAT	4.7 to 241.1	71.8	4.6	46.3
AUC 2	46	Maximum HEAT to HEAT end	10.4 to 216.3	90.3	7.4	49.9
AUC 3	46	HEAT start to HEAT end	44.5 to 294.6	167.7	9.3	63.2
Other						
Baseline VTp	131	Average of up to 20 h of recorded data beginning 7 to 33 h after PGF_2α_ administration	37.9 to 39.3	38.5	0.02	0.2
Baseline duration	131	PGF_2α_ to HEAT start	26.2 to 57.6	42.7	0.6	6.4

^1^Number of observations.

^2^VTp: Vaginal Temperature.

^3^HEAT start was defined as the first time vaginal temperature was 0.1 °C above baseline.

^4^HEAT end was defined as the time when a cow returned to baseline vaginal temperature.

^5^Rate of change equals the change in vaginal temperature divided by the change in time.

### iButton temperature verification

Ten iButtons were randomly selected for comparison of temperature readings to a digital temperature probe (GLA M900 thermometer; 7.6 cm right-angled probe; ±0.1 °C accuracy; GLA Agricultural Electronics, San Luis Obispo, CA) and mercury thermometer. iButtons were affixed to a blank CIDR as described above before submerging in the water contained within glass beakers placed in a 38.0 °C water bath (GP500 water bath; NESLAB Instruments, Inc., Newlington, NH, USA). After a 12 h acclimation period, iButton temperature readings were 37.7 ± 0.01 °C compared to 37.8 °C for the digital probe, and 38.0 °C for the mercury thermometer.

### Blood collection and serum hormone assays

Blood samples were collected from the coccygeal vein or artery at different times depicted in Figure 1A (heifers) and Figure 1B (cows). For heifers, blood samples were taken at CIDR insertion, PGF_2α_ administration, first standing mount, and 11.9 ± 0.1 h after first standing mount. For cows, blood samples were taken at PGF_2α_ administration and 63.4 ± 0.6 h (first year) and 53.6 ± 1.0 h (second year) after PGF_2α_ administration. Serum was stored at −80 °C until hormone analyses. Serum estradiol (**E2**) was determined by radioimmunoassay (Kirby et al., 1997). Sensitivity of assay was 1.03 pg/mL; intra- and inter-assay CVs were 3.2% and 8.2%, respectively, for the heifer study, and 3.3% and 9.3%, respectively, for the cow study. Serum progesterone (**P4**) concentrations were measured using an ImmuChem Double Antibody Radioimmunoassay Kit (MP Biomedicals, LLC, Orangeburg, NY, USA). Sensitivity of assay was 0.11 ng/mL; intra- and inter-assay CVs were 4.5% and 8.4%, respectively, for the heifer study and 4.4% and 8.3%, respectively, for the cow study.

### Statistical analyses

Analyses were conducted using SAS 9.4 (SAS Institute, Cary, NC, USA). Data were checked for normality using Shapiro–Wilk. Independent variables, listed in Supplementary Table 1 for heifers and Supplementary Table 2 for cows, were included in the models if deemed potentially impactful in an initial simple linear regression (*P* < 0.2). Data from heifers and cows were analyzed separately. Best fit hierarchical linear regression models were determined for continuous dependent variables using backward manual selection specifically taking low Akaike Information Criterion (**AIC**), low −2 Res Log Likelihood, and *R*^2^ values into consideration (PROC MIXED, SAS 9.4). For categorical dependent variables, logistic regression was used to determine the change in log odds of group membership for the dependent variable. Best-fit models were determined by taking low −2 Pseudo Res Log Likelihood and high generalized Chi-square values into consideration (PROC GLIMMIX, SAS 9.4). For both continuous and categorical dependent variables, *R*^2^ was calculated by dropping random effects since it is not produced by mixed model software. Heifer models included the random effects study year and first mount date nested within study year. Cow models included the random effect location by HEAT start date nested within year. In both studies, the effect of THI on vaginal temperatures during the period before and after maximum vaginal temperature was analyzed in females that displayed HEAT using a mixed model with repeated measures over time (PROC MIXED, SAS 9.4). Differences between common HEAT and other variables in heifers and cows were analyzed using mixed models with the fixed effect of study (i.e., study one [heifers] vs. study two [cows]) and the random effect of first mount or HEAT start date nested within year (PROC MIXED, SAS 9.4). Data are presented as least squares means ± standard error. Significant differences were denoted when a *P* value ≤ 0.05 unless otherwise noted.

## Results

### Study one: HEAT in beef heifers

**Figure 2.**
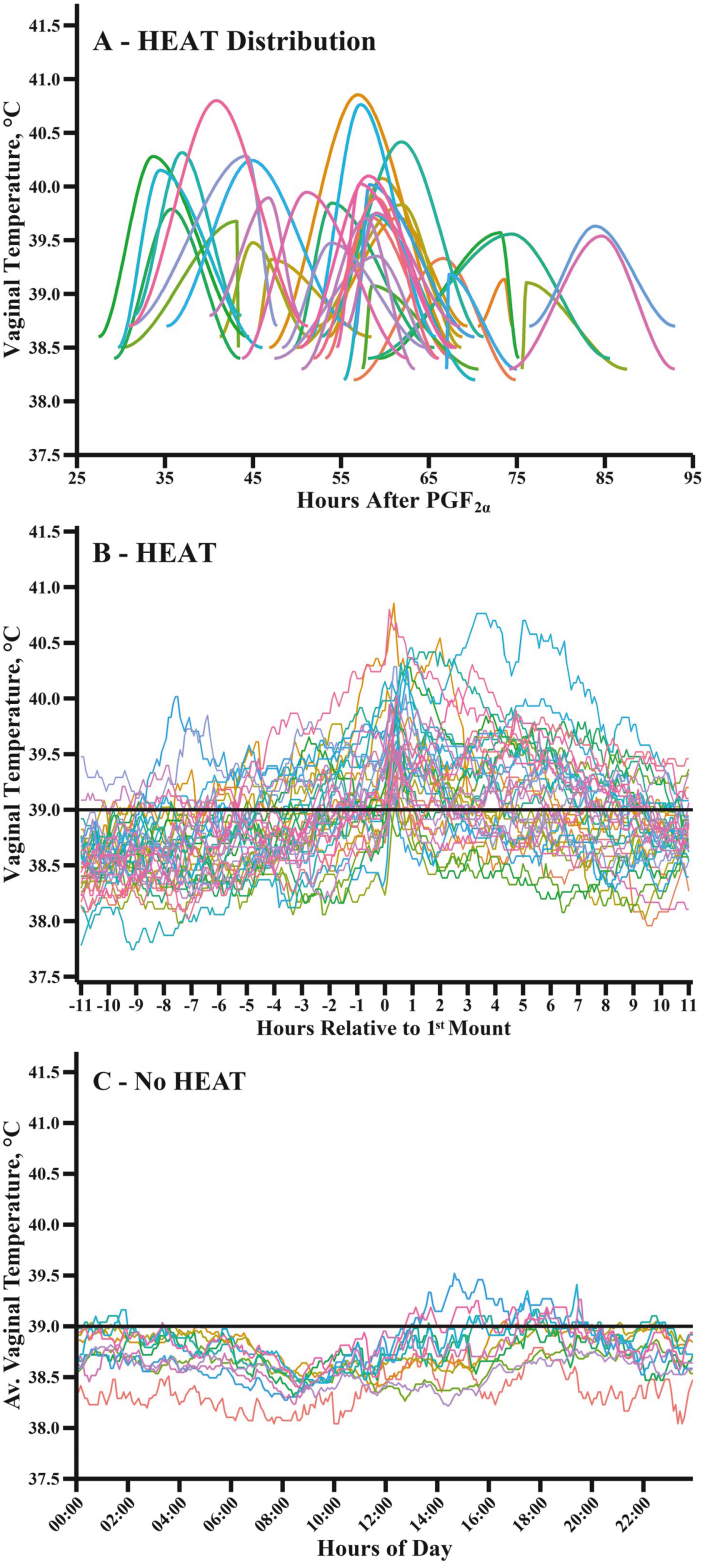
Distribution of HEAT start, maximum HEAT, and HEAT end in individual heifers after prostaglandin F__2_⍺_ (PGF__2_⍺_) administration (panel A). Varying levels of HEAT in individual heifers where vaginal temperature was plotted every 5-min in the 11 h before and the 11 h after the first standing mount (panel B). Vaginal temperature of comingled heifers that did not stand to be mounted (no HEAT) when plotted every 5-min and averaged over study days (panel C). The black line in panels B and C highlights the extent vaginal temperatures were above 39.0 °C which was the lowest maximum HEAT in heifers.

Distribution of the start of heifer HEAT ranged from 27.5 to 76.4 h after PGF_2α_ administration (Figure 2A). HEAT during the 11 h before and after a heifer first stood to be mounted varied considerably among individual animals (Figure 2B). The vaginal temperature of comingled heifers not standing to be mounted by others (i.e., No HEAT) is shown in Figure 2C. Baseline, the first-mount vaginal temperature, THI when the first mount occurred, maximum HEAT, HEAT length, and length of time HEAT was ≥39.5 and 40 °C are provided for heifers with complete data in Table 3. For this subset (*n* = 38), maximum HEAT ranged from 39.0 to 40.8 °C and averaged 39.8 °C. Interestingly, 13 out of the 38 heifers having a complete HEAT curve (34.2%) had maximum vaginal temperatures of ≥40.0 °C when thermoneutral conditions existed. Remarkably, the time heifers experienced HEAT ≥40 °C ranged from 0.1 to 6.5 h. Four of the 13 heifers remained ≥40 °C for 3.0, 3.4, 4.8 and 6.5 h, respectively. Twenty-nine heifers had a maximum temperature of ≥39.5 °C (76.3% of total). Further, the time heifers remained ≥39.5 °C ranged from 0.1 to 9.4 h.

**Table 3.** HEAT max, duration, and time at or above 39.5 and 40.0 °C in beef heifers

ID	Baseline VTp^1^ (°C)	First mount VTp (°C)	THI At the first mount	Max VTp (°C)	HEAT length (h)	≥39.5 °C (h)	≥40.0 °C (h)
35	38.5	40.4	73.1	40.8	21.3	4.7	3.4
30	38.7	40.3	59.2	40.8	20.3	9.4	4.8
18	38.6	39.0	73.1	40.7	14.3	9.3	6.5
14	38.6	40.0	68.0	40.4	18.6	7.4	3.0
15	38.8	39.7	63.4	40.3	12.3	5.8	0.5
10	38.6	39.2	64.2	40.2	17.1	6.6	1.2
23	38.7	39.7	57.7	40.2	17.0	4.9	0.3
19	38.7	39.5	57.7	40.2	21.0	6.8	0.3
17	38.5	40.2	64.2	40.1	16.4	4.0	0.6
4	38.6	39.9	68.0	40.0	15.3	2.3	0.1
29	38.5	39.0	70.4	40.0	17.4	3.1	0.2
49	38.5	39.0	71.5	40.0	13.6	3.3	0.1
21	38.6	39.7	59.6	40.0	13.7	4.3	0.1
28	38.4	39.5	71.1	39.9	18.9	1.5	-
31	38.4	38.8	73.1	39.8	14.3	5.9	-
26	38.8	39.4	57.0	39.8	10.3	3.8	-
37	38.5	39.1	63.4	39.8	18.8	1.6	-
41	38.5	39.0	75.0	39.8	16.0	0.2	-
48	38.7	39.3	68.8	39.7	12.9	0.7	-
1	38.4	39.4	68.0	39.7	14.6	1.2	-
12	38.4	38.9	71.0	39.7	14.3	4.3	-
16	38.2	39.4	68.1	39.7	14.9	2.0	-
36	38.7	39.3	66.7	39.7	17.2	2.3	-
47	38.3	38.8	75.4	39.7	12.9	0.1	-
8	38.5	39.1	69.8	39.6	13.3	2.2	-
22	38.7	39.5	55.4	39.6	16.6	2.3	-
27	38.3	39.1	53.0	39.5	18.8	0.1	-
11	38.4	38.9	57.8	39.5	15.9	0.1	-
13	38.4	38.8	57.8	39.5	27.5	0.1	-
6	38.6	39.1	57.9	39.4	10.3	-	-
24	38.5	39.3	72.2	39.4	16.6	-	-
25	38.4	38.9	73.1	39.3	18.6	-	-
2	38.2	39.0	59.6	39.3	18.4	-	-
5	38.6	38.8	59.7	39.3	13.2	-	-
20	38.3	38.4	59.5	39.1	8.0	-	-
3	38.7	38.7	57.8	39.1	3.9	-	-
7	38.3	38.2	64.2	39.1	11.9	-	-
9	38.3	38.8	59.6	39.0	13.2	-	-

^1^Vaginal temperature.

#### Influence of ambient conditions on HEAT

Ambient conditions and the time-of-day heifers first stood to be mounted during years 1 and 2 are shown in Supplementary Figure 1. In the 11 h leading up to maximum HEAT, vaginal temperature increased (*P *= 0.0001; Supplementary Figure 2A). Conversely, in the 11 h after maximum HEAT, vaginal temperature decreased (*P *= 0.0001; Supplementary Figure 2C). Vaginal temperature during the 11 h before maximum HEAT was not affected by THI (*P* = 0.5146; Supplementary Figure 2B) nor was it convincingly impacted by THI in the 11 h after maximum HEAT (e.g., when THI was ≤50 vaginal temperatures were similar to those recorded when THI was ≥71; Supplementary Figure 2D).

#### Other factors significantly associated with different HEAT-dependent variables

The first-mount vaginal temperature was associated with baseline temperature and duration, and proestrus length which was defined as the length of time from PGF administration to the first mount (Table 4). Per each 1 °C increase in baseline, vaginal temperature at first mount increased by 0.98 °C (*P* = 0.0280). When baseline duration increased by 1-h, vaginal temperature at first mount decreased by 0.06 °C (*P* = 0.0028). Interestingly, per each 1 h increase in proestrus length, vaginal temperature at first mount increased by 0.04 °C (*P* = 0.0121). When expressed as a change from baseline, the first-mount vaginal temperature remained associated with baseline duration (*P* = 0.0022) and proestrus length (*P* = 0.0108).

**Table 4.** Associated factors with HEAT variables in heifers

HEAT dependent variables	AIC	−2 Res log likelihood	*R* ^2^	Associated independent variables	*P* value	Slope
Vaginal temperature (°C)						
First-mount vaginal temperature	53.8	51.8	0.42	Baseline VTp^1^	0.0280	0.98
				Baseline duration	0.0028	−0.06
				Proestrus length	0.0121	0.04
First mount (baseline change)	53.9	51.9	0.28	Baseline duration	0.0022	−0.06
				Proestrus length	0.0108	0.04
Maximum HEAT	42.9	40.9	0.48	CIDR_in_ E2	0.0115	0.10
				Baseline Duration	0.0017	−0.02
Maximum HEAT (baseline change)	43.6	41.6	0.34	CIDR_in_ E2	0.0147	0.09
				Baseline duration	0.0020	−0.01
Duration, h						
Maximum HEAT to HEAT end	198.7	194.7	0.28	Follicle Growth (CIDR_out_ to iButton_out_)	0.0030	0.77
				Baseline VTp	0.0432	−5.46
HEAT length	210.6	208.6	0.11	CIDR_in_ E2	0.0455	0.84
Rate of temperature change, (°C/h)						
HEAT start to the first mount	−72.1	−76.1	0.05	Baseline VTp	0.0067	−0.21
Maximum HEAT to HEAT end	−52.3	−56.3	0.46	CIDR_in_ P4	0.0153	−0.01
				Follicle growth (CIDR_out_ to iButton_out_)	0.0050	0.02
				Baseline VTp	0.0004	−0.32
Area under the heat curve						
HEAT start to maximum HEAT	350.3	348.3	0.30	Baseline duration	0.0016	−1.18
First mount to HEAT end	374.2	370.2	0.37	CIDR_in_ E2	0.0017	9.63
				Follicle growth (CIDR_out_ to iButton_out_)	0.0002	10.47
				Baseline VTp	0.0018	−97.95
Maximum HEAT to HEAT end	396.2	390.2	0.20	Follicle growth (CIDR_out_ to iButton_out_)	0.0005	10.75
HEAT start to HEAT end	385.7	383.7	0.29	CIDR_in_ age	0.0373	0.40
				Baseline duration	0.0083	−1.47

Vaginal temperature change the first mount to maximum HEAT, duration of HEAT start to the first mount, HEAT start to maximum HEAT, the first mount to maximum HEAT, the first mount to HEAT end, and rate of change HEAT start to maximum HEAT, and the first mount to maximum HEAT were not significantly associated with any of the independent variables tested. Area under the curve from HEAT start to the first mount was not significantly associated with any of the independent variables tested.

^1^Vaginal temperature.

Vaginal temperature at maximum HEAT was associated with estradiol level at CIDR insertion and baseline duration (Table 4). Per each 1 pg/mL increase in estradiol at CIDR insertion, maximum HEAT increased by 0.10 °C (*P* = 0.0115). Also, per each 1 h increase in baseline duration, maximum HEAT decreased by 0.02 °C (*P* = 0.0017). When expressed as a change from baseline, maximum HEAT remained associated with estradiol level at CIDR insertion (*P* = 0.0147) and baseline duration (*P* = 0.0020).

The number of hours from maximum HEAT to HEAT end was associated with the change in ovulatory follicle size from PGF_2α_ administration to iButton removal (~11.9 ± 0.1 h after first mount) and baseline vaginal temperature (Table 4). Per each 1 mm increase in ovulatory follicle growth, the number of hours from maximum HEAT to HEAT end increased by 0.77 h (*P* = 0.0030). Further, each 1 °C increase in baseline was associated with a 5.46 h decrease in the number of hours from maximum HEAT to HEAT end (*P* = 0.0432). Interestingly, per each 1 °C increase in baseline, there was an 82.5% increase in the odds that a heifer returned to baseline within 11.9 ± 0.1 h of first standing mount (*P* = 0.0428). Length of HEAT was only associated with estradiol level at CIDR insertion. Per each 1 pg/mL increase in estradiol at CIDR insertion, HEAT length increased by 0.84 h (*P* = 0.0455).

Rate of change in vaginal temperature from HEAT start to the first mount was negatively associated with baseline vaginal temperature (Table 4). Per each 1 °C increase in baseline vaginal temperature, rate of the temperature increase was 0.21 °C/h slower (*P* = 0.0067). The rate of vaginal temperature change from maximum HEAT to HEAT end was associated with progesterone level at CIDR insertion, ovulatory follicle growth, and baseline vaginal temperature (Table 4). Per each one ng/mL increase in progesterone at CIDR insertion, rate of the temperature decrease was 0.01 °C/h faster (*P* = 0.0153). Per each one mm increase in ovulatory follicle growth, rate of the temperature decrease was 0.02 °C/h slower (*P* = 0.0050). Per each 1 °C increase in baseline vaginal temperature, rate of the temperature decrease was 0.32 °C/h faster (*P* = 0.0004).

Factors associated with different areas under the HEAT curve are provided in Table 4. Area under the HEAT curve from HEAT start to maximum HEAT (**AUC2**) was negatively associated with baseline duration. Per each 1 h increase in baseline duration, AUC2 decreased by 1.18 units (*P* = 0.0016). Area under the HEAT curve from first mount to HEAT end (**AUC3**), was associated with estradiol level at CIDR insertion, ovulatory follicle growth, and baseline VTp. Per each 1 pg/mL increase in estradiol at CIDR insertion, AUC3 increased by 9.63 units (*P* = 0.0017). Per each one mm increase in ovulatory follicle growth, AUC3 increased by 10.47 units (*P* = 0.0002). In contrast, per each 1 °C increase in baseline VTp, AUC3 decreased by 97.95 units (*P* = 0.0018). Area under the HEAT curve from maximum HEAT to HEAT end (**AUC4**) was positively associated with ovulatory follicle growth. Per each one mm increase in ovulatory follicle growth, AUC4 increased by 10.75 units (*P* = 0.0005). Area under the entire HEAT curve (**AUC5**) was negatively associated with baseline duration. Per each hour increase in baseline duration, AUC5 decreased by 1.47 units (*P* = 0.0083).

### Study two: HEAT in suckled beef cows

**Figure 3.**
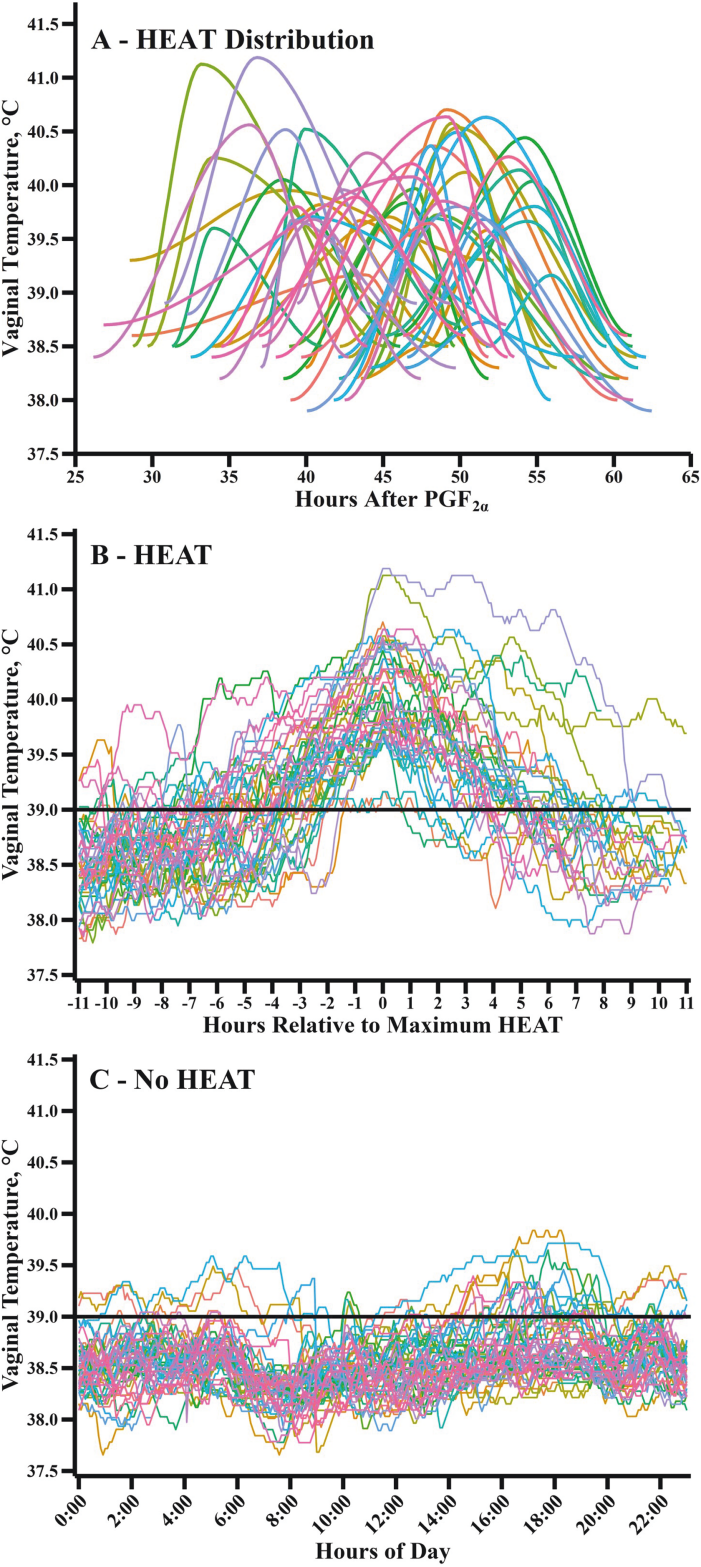
Distribution of HEAT start, maximum HEAT, and HEAT end in individual cows after prostaglandin F__2_⍺_ (PGF__2_⍺_) administration (panel A). Varying levels of HEAT in individual cows where vaginal temperature was plotted every 5-min in the 11 h before and the 11 h after maximum HEAT (panel B). Vaginal temperature of a random subset of comingled cows equally distributed each year and location with an EP score of 1 (no HEAT) by 63.4 ± 0.6 h (first year) or 53.6 ± 1.0 h (second year) after PGF_2α_ administration was plotted every 5-min and averaged over study days (panel C). The black line in panels B and C highlights maximum HEAT exceeded 39.0 °C in all estrual cows.

Distribution of the start of cow HEAT ranged from 26.2 to 57.6 h after PGF_2α_ administration (Figure 3A). HEAT during the 11 h before and after a cow reached maximum vaginal temperature varied considerably among individual animals (Figure 3B). The vaginal temperatures of a random subset of comingled cows, equally distributed between each year and location, with EP scores of 1 (i.e., no HEAT) are shown in Figure 3C. For cows with complete HEAT data, Table 5 provides information on baseline, maximum HEAT, THI at maximum HEAT, study year, location, HEAT length, length of time HEAT episodes ≥39.5, ≥40, and ≥41 °C. Maximum cow HEAT ranged from 39.1 to 41.1 °C and averaged 40.1 °C in this subset of cows (*n* = 46). Two out of the 46 cows (4.3%) remained at or above 41 °C for 1.2 and 3.6 h. Twenty-three cows reached maximum HEAT temperatures ≥ 40 °C. The range of time that HEAT was ≥40 °C was 0.1 to 10.0 h. The majority of the cows (*n* = 44; 95.7%) had maximum temperatures of ≥39.5 °C. Cows remained at or above 39.5 °C for 0.4 to 24.8 h.

**Table 5.** HEAT max, duration, and time at or above 39.5, 40.0, and 41.0 °C in suckled beef cows

ID	Baseline VTp^1^ (°C)	Max VTp (°C)	THI at max	Yr	Loc	HEAT length (h)	≥39.5 °C (h)	≥40.0 °C (h)	≥41.0 °C (h)
27	38.9	41.1	57.6	2	3	16.4	12.1	10.0	3.6
58	38.5	41.1	61.7	2	3	16.5	9.8	8.3	1.2
94	38.2	40.7	67.6	1	2	17.8	6.9	3.8	-
22	38.4	40.6	59.0	1	2	20.0	8.7	5.8	-
34	38.4	40.6	63.7	1	2	19.7	15.9	7.1	-
10	38.3	40.5	63.7	1	2	11.5	6.3	3.4	-
31	38.4	40.5	50.8	2	3	16.6	9.0	5.2	-
99	38.4	40.5	63.7	1	2	18.8	7.3	6.1	-
26	38.8	40.5	53.2	2	1	11.9	9.4	5.8	-
73	38.7	40.5	48.7	2	1	12.7	9.2	8.3	-
82	38.0	40.4	62.7	1	2	14.1	6.2	4.0	-
64	38.6	40.4	47.6	1	1	15.4	11.0	9.1	-
93	38.0	40.3	67.6	1	2	21.3	4.0	2.2	-
3	38.9	40.3	57.1	1	1	6.2	2.6	1.0	-
33	38.5	40.3	44.3	2	3	15.2	6.8	2.4	-
5	38.6	40.2	48.7	1	1	14.6	9.8	4.2	-
57	38.5	40.2	61.7	2	3	19.3	14.4	3.4	-
38	38.4	40.2	57.5	2	3	15.1	7.1	2.4	-
98	38.5	40.1	62.7	1	2	13.8	5.2	0.8	-
14	38.6	40.1	47.6	1	1	14.6	6.0	0.7	-
1	38.5	40.0	47.7	2	3	14.7	6.2	0.1	-
89	38.7	40.0	58.9	2	2	26.2	24.8	1.6	-
69	38.5	40.0	57.6	1	1	12.8	5.7	0.3	-
52	39.3	39.9	47.7	2	3	23.7	5.3	-	-
87	38.9	39.9	56.5	2	2	10.0	7.6	-	-
60	38.5	39.9	49.1	2	1	10.8	2.5	-	-
88	38.0	39.8	67.6	1	2	18.8	4.9	-	-
39	38.6	39.8	45.9	2	3	13.4	6.8	-	-
25	37.9	39.8	62.8	1	2	22.4	4.1	-	-
77	38.3	39.8	49.2	1	2	17.3	4.3	-	-
8	38.5	39.8	45.2	2	3	15.4	5.8	-	-
36	38.5	39.8	46.8	2	3	9.5	4.2	-	-
61	38.2	39.8	42.3	2	1	13.3	3.4	-	-
11	38.2	39.7	67.6	1	2	16.7	3.3	-	-
51	38.5	39.7	62.3	1	2	18.0	1.4	-	-
78	38.4	39.7	53.2	1	2	25.2	0.8	-	-
86	38.4	39.6	55.3	1	2	11.5	3.5	-	-
15	38.3	39.6	45.1	1	1	17.6	3.2	-	-
74	38.2	39.6	54.1	1	1	17.2	2.3	-	-
28	38.3	39.6	47.7	2	3	12.7	2.7	-	-
29	38.2	39.6	50.8	2	3	13.1	1.8	-	-
95	38.3	39.6	57.7	2	2	12.6	1.2	-	-
45	38.4	39.6	63.1	2	3	12.2	0.4	-	-
71	38.5	39.6	55.0	2	2	9.8	0.5	-	-
16	38.5	39.1	47.7	1	2	7.8	-	-	-
47	38.6	39.1	45.2	2	3	18.2	-	-	-

^1^Vaginal temperature.

#### Influence of ambient conditions on HEAT

Ambient conditions along with the time-of-day maximum HEAT occurred during year 1 (panels A–B) and year 2 (panels C–E) per each location are shown in Supplementary Figure 3. Vaginal temperature increased in the hours leading up to maximum HEAT (*P *= 0.0001; Supplementary Figure 4A) and decreased in the hours thereafter (*P *= 0.0001; Supplementary Figure 4C). In the hours approaching maximum HEAT, vaginal temperature was affected by THI. Specifically, when THI ranged from ≤45 to ≥66, vaginal temperature increased by 0.3 °C (*P *= 0.0020; Supplementary Figure 4B). However, in the 11 h after maximum HEAT, THI did not affect vaginal temperature (*P *= 0.1802; Supplemental Figure 4D).

#### Other factors significantly associated with different HEAT-dependent variables

Maximum HEAT vaginal temperature was positively associated with baseline vaginal temperature (Table 6). Per each 1 °C increase in baseline, maximum HEAT increased by 0.62 °C (*P* = 0.0031).

**Table 6.** Associated factors with different HEAT variable in suckled beef cows

HEAT-dependent variables	AIC	−2 Res log likelihood	*R* ^2^	Associated independent variables	*P* value	Slope
Vaginal temperature (°C)						
Maximum HEAT	128.9	124.9	0.09	Baseline VTp^1^	0.0031	0.62
Duration, h						
HEAT start to maximum HEAT	529.7	525.7	0.22	Baseline duration	0.0001	−0.34
HEAT length	247.0	243.0	0.48	Weight	0.0042	0.02
				Follicle growth (CIDR_out_ to iButton_out_)	0.0032	0.54
				Baseline duration	0.0001	−0.47
Rate of temperature change (°C/h)						
HEAT start to maximum HEAT	−77.9	−79.9	0.33	Hair coat score	0.0001	−0.08
				Baseline duration	0.0455	0.01
Maximum HEAT to HEAT end	−71.1	−73.1	0.09	CIDR_out_ P4	0.0486	−0.04
Area under the curve						
HEAT start to maximum HEAT	1013.6	1009.6	0.21	Baseline duration	0.0001	−3.79
HEAT start to HEAT end	488.6	486.6	0.22	Age	0.0043	11.92
				Baseline duration	0.0062	−4.39

Maximum HEAT (baseline change), duration of maximum HEAT to HEAT end (h), and area under the HEAT curve from maximum HEAT to HEAT end were not significantly associated with any of the independent variables tested.

^1^VTp: Vaginal Temperature.

The number of hours from HEAT start to maximum HEAT was negatively associated with baseline duration (Table 6). Per each hour increase in baseline duration, the number of hours from HEAT start to maximum HEAT decreased by 0.34 h (*P* = 0.0001). Length of HEAT was associated with weight, the change in ovulatory follicle size from PGF_2α_ administration to GnRH administration, and baseline duration (Table 6). Per each 1 kg increase in weight, HEAT length increased by 0.02 h (*P* = 0.0042). Per each 1 mm increase in ovulatory follicle growth, HEAT length increased by 0.54 h (*P* = 0.0032). Further, per each hour increase in baseline duration, HEAT length decreased by 0.47 h (*P* = 0.0001).

The rate of change in vaginal temperature from HEAT start to maximum HEAT was associated with baseline duration and haircoat score (Table 6). Per each hour increase in baseline duration, rate of the temperature increase was 0.01 °C per hour faster (*P* = 0.0455). Per each unit increase in hair coat score, rate of the temperature increase was 0.08 °C/h slower (*P* = 0.0001). The rate of vaginal temperature change from maximum HEAT to HEAT end was associated with progesterone level at PGF_2α_ administration (Table 6). Per each one ng/mL increase in progesterone at PGF_2α_ administration, rate of the temperature decrease was 0.04 °C per h faster (*P* = 0.0486).

Area under the HEAT curve from HEAT start to maximum HEAT (AUC1) was negatively associated with baseline duration (Table 6). Per each hour increase in baseline duration, AUC1 decreased by 3.79 units (*P* = 0.0001). The area under the entire HEAT curve (AUC3) was associated with cow age and baseline duration (Table 6). Per each year increase in age, AUC3 increased by 11.92 units (*P* = 0.0043). Per each hour increase in baseline duration, AUC3 decreased by 4.39 units (*P* = 0.0062).

### Comparison of HEAT and other variables in heifers and suckled beef cows

Maximum HEAT and change from baseline, number of hours from both HEAT start to maximum, and maximum to HEAT end, HEAT length, rate of change from HEAT start to maximum HEAT, areas under HEAT curve from start to maximum and maximum to HEAT end, and baseline vaginal temperature and duration were similar in heifers and suckled beef cows (Table 7). Interestingly, cows had a faster temperature decrease from maximum HEAT to HEAT end (*P *= 0.0012) compared to heifers. In addition, total area under the HEAT curve from HEAT start to HEAT end was greater for cows than heifers (*P *= 0.0571). Heifers had higher levels of progesterone at PGF_2α_ administration than cows (*P *= 0.0097). At PGF_2α_ administration, estradiol levels were similar (*P *= 0.1938) despite cows having larger ovulatory follicle size (*P *= 0.0002) than heifers.

**Table 7.** Comparison of common HEAT and other variables in heifers and cows

HEAT Variables	Heifer	Cow	*P* value
Vaginal temperature (°C)			
Maximum HEAT	39.9 ± 0.1	40.0 ± 0.1	0.6681
Maximum HEAT (baseline change)	1.5 ± 0.1	1.5 ± 0.1	0.6331
Duration (h)			
HEAT start to maximum HEAT	6.9 ± 1.2	7.1 ± 0.9	0.9036
Maximum HEAT to HEAT end	9.6 ± 0.7	7.7 ± 0.7	0.0741
HEAT length	15.5 ± 0.8	15.4 ± 0.7	0.9394
Rate of temperature change (°C/h)			
HEAT start to maximum HEAT	0.21 ± 0.03	0.28 ± 0.02	0.1086
Maximum HEAT to HEAT end	−0.15 ± 0.02	-−0.22 ± 0.02	0.0012
Area under the curve			
HEAT start to maximum HEAT	49.3 ± 13.9	72.2 ± 9.6	0.2119
Maximum HEAT to HEAT end	76.1 ± 10.0	83.8 ± 10.3	0.6071
HEAT start to HEAT end	117.1 ± 13.5	158.7 ± 12.3	0.0571
Baseline			
Vaginal temperature (°C)	38.5 ± 0.05	38.5 ± 0.03	0.7064
Duration (h)	48.0 ± 5.1	43.2 ± 3.3	0.4491
Other variables			
CIDR_out_ P4 (ng/mL)	6.0 ± 1.0	2.3 ± 0.7	0.0097
CIDR_out_ E2 (pg/mL)	4.4 ± 1.4	6.7 ± 1.0	0.1938
CIDR_out_ Ov1 (mm)	10.4 ± 0.5	13.2 ± 0.3	0.0002

Data are presented as least square means ± SEM.

## Discussion

A novelty of this research relates to the characterization of the varying levels of HEAT in beef heifers and suckled beef cows at the onset of a typical breeding season where ambient conditions were predominantly thermoneutral and pregnancy outcomes per artificial insemination are expected to exceed 50%. Continuous measurements taken at 5-min intervals, and without averaging, not only provided a more accurate visualization of a pointed HEAT curve and better estimate of maximum HEAT but also allowed for examining the length of time estrual heifers and cows may remain hyperthermic. To this end, the majority of beef heifers (81.6%) and suckled cows (94.0%) met the clinical definition of hyperthermia [≥39.5 °C; (Constable et al., 2016)]. Surprisingly, 42.9% of heifers and 49.0% of cows had vaginal temperatures ≥40 °C. In fact, one heifer and five cows’ maximum HEAT exceeded 41.0 °C. The average maximum HEAT for beef heifers and suckled cows in the current study was 39.9 and 40.0 °C which is similar to maximum HEAT values reported by two others (Cooper-Prado et al., 2011; Kim et al., 2023). Depending on the animal, hyperthermia persisted from 0.1 to 9.4 h in heifers (3.4 ± 2.7 h) and 0.4 to 24.8 h in suckled cows (6.2 ± 4.6 h). Interestingly, 42.9% of heifers and 49.0% of cows had vaginal temperatures ≥40 °C which persisted up to 6.5 (1.6 ± 2.1) and 10 (4.2 ± 1.7) h, respectively. Total time ≥41.0 °C was 1.2 and 3.6 h for the 2 cows with complete HEAT data. Before reaching 41 °C, the total time above 40 °C for these 2 cows was 8.3 and 10 h, respectively. It is worth noting that both cows whose maximum HEAT exceeded 41 °C and were at 40 °C for an extended time, were determined pregnant after FTAI.

Average HEAT length was 15.5 h in heifers and cows which is longer than when the start of estrus is defined by the first mount (Van Eerdenburg et al., 1996; Kerbrat and Disenhaus, 2004; Yoshida and Nakao, 2005; Silper et al., 2015). In heifers, the amount of time after HEAT onset until the first standing mount was 5.9 ± 0.6 h. Walking activity alone or activity associated with estrus relates well to increased body temperatures (Murray and Yeates, 1967; Burnett et al., 2020). Interestingly, heifers reached maximum HEAT, on average 1.2 ± 0.5 h after the first standing mount. During this time, 74.1% of heifers where mounting activity was available, experienced 0 to 1 mount. Typically, the LH surge occurs soon after the first mount (Chenault et al., 1976; Rajamahendran and Taylor, 1991; Aungier et al., 2015); whereas peak HEAT occurs shortly after the LH surge (Rajamahendran et al., 1989; Mosher et al., 1990; Fisher et al., 2008; Higaki et al., 2019).

Although many HEAT variables were similar among heifers and cows, suckled beef cows had a more pronounced HEAT (larger total AUC and remained at or above 39.5 to 40 °C twice as long as heifers). Suckled beef cows exhibiting estrus may not be able to thermoregulate as well as heifers because of the extra metabolic demands associated with lactation (Kadzere et al., 2002; Cartwright et al., 2023) and possible stress being with calf. Another interesting difference among heifers and cows, heifers returned to baseline temperature at a slower rate. In retrospect, more frequent monitoring and handling of heifers may have contributed to their slower return to baseline after reaching maximum HEAT.

Toward identifying factors related to magnitude and persistence, ambient conditions had no impact on HEAT in heifers and were marginally impactful on cows. Because THI was predominantly in the thermoneutral range (Kadzere et al., 2002) this is unsurprising. Vaginal temperature of suckled beef cows increased by 0.3 °C when THI was ≥66. A minor effect on HEAT is to be expected given that lactating cows are prone to thermoregulatory challenges as ambient conditions change (Kadzere et al., 2002; Cartwright et al., 2023). Although significant, overall impact of THI was marginal considering suckled beef cows experienced a maximum HEAT-related temperature change from baseline of 1.5 ± 0.05 °C.

Regarding other identified factors relating to different aspects of HEAT, baseline vaginal temperature and duration were the most recurrent. A higher baseline temperature was associated with a higher first mount temperature in heifers, and maximum HEAT in both heifers and cows. Although it is intuitive that cattle with higher baseline temperatures have higher first mount and maximum HEAT, it was interesting that HEAT was shorter and of less magnitude. Whether this provides a protective mechanism to prevent cattle from reaching harmful temperatures for extended time periods remains unclear. In heifers with a longer baseline duration, vaginal temperature at the first mount and maximum HEAT were lower. In cows, a longer baseline duration related to 1) a shorter time from HEAT start to maximum HEAT, 2) a shorter HEAT length, and 3) vaginal temperature increased at a faster rate from HEAT start to maximum HEAT. In both heifers and cows, longer baseline duration was associated with a smaller AUC from HEAT start to maximum HEAT and HEAT start to HEAT end.

Increased activity associated with displaying secondary signs of impending estrus (on average an additional 5.9 ± 0.6 h after HEAT start but before the first mount) likely explains why a longer proestrus length was associated with higher vaginal temperature at the first standing mount. In instances where proestrus length was five or more hours from HEAT start to the first standing mount, vaginal temperatures at the first standing mount were higher than those with less than 5 h from HEAT start to the first mount (*P* = 0.0043; data not shown).

Growth of the presumed ovulatory follicle after PGF_2α_ administration was associated with several HEAT variables in heifers and cows. Rodrigues et al. (2018) reported that beef cows housed on pasture, with higher net physical activity (pedometer with EP) in the 2 d before FTAI, had a larger follicle than cows with lower net physical activity. Functional significance of these findings remains unclear. Interestingly, estradiol levels at any time after CIDR insertion were not related to any aspect of HEAT. In retrospect, this is unsurprising because estradiol levels at or around the time of the first mount have been reported to have a weak to no relationship with mounting intensity of dairy heifers and cows (De Silva et al., 1981; Glencross et al., 1981) or estrual activity measured by pedometers or accelerometers (Aungier et al., 2015; Madureira et al., 2015, 2019).

Higher concentrations of progesterone at CIDR removal/PGF_2α_ administration in heifers and cows were associated with a more rapid decline in vaginal temperature from maximum HEAT to HEAT end. Although interesting, these findings are difficult to explain because progesterone concentrations decrease to less than 1 ng/mL after PGF_2α_ administration (Ginther et al., 2007; Stevenson, 2008). Davidge et al. (1987) observed a linear decrease in the number of standing mounts when administering differing amounts of progesterone (100, 300, or 500 mg) before estradiol administration to induce estrus behavior. Depending on the extent standing to be mounted slows the temperature decrease after maximum HEAT, it is interesting to speculate that heifers receiving less mounts may return to baseline vaginal temperature at a faster rate.

When striving to better understand study outcomes, the amount of HEAT variation explained or not, by associated factors was considered. Associated factors including follicle size, estradiol and progesterone levels, ambient conditions, and other animal-related variables (i.e., age, weight, hair coat score, baseline temperature, and duration) only explained only a small amount of HEAT variation (*R*^2^ of best-fit models ranged from 0.05 to 0.48). Only four HEAT variable models had *R*^2^ values ≥ 0.40. In other words, when using *R*^2^ values to indicate the extent to which the factors listed above explain the variance observed in HEAT, 52% to 95% of HEAT variation was not explained. Furthermore, 11 out of the 30 HEAT variables tested were not associated with any of the independent variables evaluated (temperature variables, *n* = 2; duration variables, *n* = 5; rate of change variables, *n* = 2; and AUC variables, *n* = 2). Mindful of this it is worth reiterating that different aspects of HEAT were characterized in heifers and cows after undergoing estrus synchronization.

Estrus synchronization effectively creates synchronous groups of estrual females that become active participants in different sexually active groups. Sakatani et al. (2016) reported that synchronized Japanese Black cows had larger temperature changes from baseline when compared to cows experiencing natural HEAT. Although level of estrous activity was not measured in the current study, it is likely contributive to HEAT. Redden et al. (1993) reported that 70.6% of estrus events detected by vaginal temperature monitoring occurred during the period when females were unrestricted in an exercise paddock. More recently, more intense estrus activity was associated with a larger maximum temperature change from baseline (Burnett et al., 2020).

## Conclusion

In conclusion, HEAT is a normal part of the periovulatory microenvironment in females exhibiting estrus. Novel outcomes described herein, however, highlight just how variable HEAT may be in individual beef heifers and suckled beef cows when occurring at the onset of a spring breeding season when thermoneutral conditions predominate. Although many aspects of HEAT were similar in heifers and cows, suckled beef cows had a pronounced HEAT that persisted longer. Interestingly, 42.9% of heifers and 49% of cows reached temperatures ≥ 40 °C which in some cases persisted up to 6.5 and 10 h, respectively. When attempting to identify factors that could explain why some females exhibiting estrus remained hot for an extended time, available animal and environmental data contributed little. Because HEAT may persist for an extended time, direct effects on fertility important components may be unavoidably impactful. The adage “too much of a good thing may be bad” likely applies to fertility-related consequences. For instance, a prolonged and direct exposure of COCs to 41 °C for the first 12 h of maturation reduces blastocyst development by as much as 42 to 65% [(in vivo hyperthermia, Putney et al., 1989); (in vitro heat stress, Edwards and Hansen, 1996; Zhandi et al., 2009)]. In contrast, direct exposure of COCs to 41 °C for 6 h hastened germinal vesicle breakdown without negative effects on meiotic progression to metaphase II (Edwards et al., 2005; Hooper et al., 2015) or blastocyst development (Ju et al., 1999; Báez et al., 2019; Rowinski et al., 2021). Whether HEAT is good, bad or matters for pregnancy is the basis of ongoing and future studies.

## Supplementary Material

skae079_suppl_Supplementary_Materials

## Abbreviations

AIC: Akaike Information Criterion
AUC: area under the curve
BS: blood sample
CIDR: controlled internal drug release
CL: corpus luteum
COC: cumulus-oocyte complexes
E2: estradiol
EP: Estrotect patch
FTAI: fixed-time artificial insemination
GnRH: gonadotropin-releasing hormone
HEAT: higher estrus-associated temperatures
LH: luteinizing hormone
P4: progesterone
PGF2α: prostaglandin F_2α_
THI: temperature humidity index
US: ovarian ultrasound
VTp: vaginal temperature
