# Supplementary material for: Magnitude and persistence of higher estrus-associated temperatures in beef heifers and suckled cows
Source: J Anim Sci. 2024 Mar 19;102:skae079. doi: 10.1093/jas/skae079 (PMC10998462; doi:10.1093/jas/skae079)
Supplement: skae079_suppl_Supplementary_Materials [file skae079_suppl_supplementary_materials.docx]

**List of Supplemental Tables**

**Supplemental Table 1.** Independent variables used to examine relationships with different higher estrus associated temperature (HEAT) variables in beef heifers

**Supplemental Table 2.** Independent variables used to examine relationships with different higher estrus associated temperature (HEAT) variables in suckled beef cows

| **Supplemental Table 1:**  Independent variables used to examine relationships with different higher estrus associated temperature (HEAT) variables in beef heifers | | | | | |
| --- | --- | --- | --- | --- | --- |
| **Variables of Interest** | **n^1^** | **Range** | **Mean** | **SEM** | **SD** |
| Age^2^ | 49 | 396.0 to 574.0 | 441.2 | 4.8 | 33.9 |
| **Progesterone,** ng/mL (P4) | | | | | |
| CIDR_in_ P4 | 49 | 0.1 to 12.4 | 3.3 | 0.4 | 2.6 |
| CIDR_out_ P4 | 49 | 1.2 to 16.4 | 6.6 | 0.6 | 4.0 |
| 1^st^ Mount P4 | 49 | 0.1 to 0.9 | 0.4 | 0.03 | 0.2 |
| Change in P4 from CIDR_in_ to CIDR_out_ | 49 | -10.3 to 12.6 | 3.2 | 0.7 | 4.7 |
| Change in P4 from CIDR_out_ to 1^st^ Mount | 49 | -15.7 to -1.0 | -6.1 | 0.6 | 4.3 |
| Change in P4 from 1^st^ Mount to iButton_out_ | 49 | -0.7 to 0.9 | -0.2 | 0.04 | 0.3 |
| **Estradiol,** pg/mL (E2) | | | | | |
| CIDR_in_ E2 | 49 | 0.8 to 12.5 | 3.2 | 0.3 | 2.4 |
| CIDR_out_ E2 | 48 | 0.9 to 9.5 | 4.0 | 0.4 | 2.7 |
| 1^st^ Mount E2 | 49 | 6.4 to 25.8 | 13.2 | 0.8 | 5.7 |
| Change in E2 from CIDR_out_ to 1^st^ Mount | 48 | 3.1 to 18.3 | 9.3 | 0.6 | 4.1 |
| **Follicle Size & Growth,** mm | | | | | |
| CIDR_out_ Ov1^3^ | 44 | 6.0 to 16.0 | 10.4 | 0.3 | 2.1 |
| 1^st^ Mount Ov1 | 48 | 10.1 to 19.7 | 13.6 | 0.3 | 2.1 |
| Ov1 Growth CIDR_out_ to 1^st^ Mount | 43 | -2.0 to 7.6 | 3.2 | 0.3 | 2.0 |
| Ov1 Growth 1^st^ Mount to iButton_out_ | 44 | -4.5 to 4.5 | 0.3 | 0.3 | 2.1 |
| Ov1 Growth CIDR_out_ to iButton_out_ | 40 | -0.6 to 7.7 | 3.4 | 0.3 | 2.0 |
| **Proestrus Length,** h | | | | | |
| PGF_2α_ to 1^st^ Mount | 49 | 30.3 to 84.3 | 51.7 | 2.0 | 14.1 |
| **Baseline Vaginal Temperature** | | | | | |
| Baseline, °C | 49 | 38.1 to 38.9 | 38.5 | 0.02 | 0.2 |
| Baseline Duration, h | 38 | 27.5 to 76.4 | 49.7 | 2.1 | 13.1 |
| ^1^Number of observations  ^2^Age, in days, at CIDR_in_  ^3^Ov1: Presumed ovulatory follicle | | | | | |

| **Supplemental Table 2:**  Independent variables used to examine relationships with different higher estrus associated temperature (HEAT) variables in suckled beef cows | | | | | |
| --- | --- | --- | --- | --- | --- |
| **Variables of Interest** | **n^1^** | **Range** | **Mean** | **SEM** | **SD** |
| Age^1^ | 100 | 2.0 to 11.0 | 4.7 | 0.2 | 2.4 |
| Weight, kg | 100 | 407.3 to 907.2 | 599.5 | 8.4 | 95.7 |
| Days Post-Partum^2^ | 100 | 34.0 to 119.0 | 79.5 | 1.7 | 20.0 |
| Calf Sex | 100 | . | . | . | . |
| Calf Birth Weight | 98 | 18.1 to 43.1 | 33.3 | 0.4 | 4.8 |
| Body Condition Score^3^ | 69 | 5.0 to 8.0 | 5.9 | 0.1 | 0.7 |
| Hair Coat Score^3^ | 69 | 1.0 to 5.0 | 3.2 | 0.1 | 0.9 |
| **Progesterone,** ng/mL (P4) | | | | | |
| CIDR_out_ P4 | 100 | 0.1 to 10.8 | 2.2 | 0.1 | 1.5 |
| Change in P4 from CIDR_out_ to FTAI^4^ | 100 | -10.7 to 0.1 | -2.0 | 0.1 | 1.5 |
| **Estradiol,** pg/mL (E2) | | | | | |
| CIDR_out_ E2 | 99 | 0.2 to 17.0 | 7.0 | 0.3 | 3.9 |
| Change in E2 from CIDR_out_ to FTAI | 98 | -7.9 to 17.2 | 2.8 | 0.5 | 5.2 |
| **Follicle Size & Growth,** mm | | | | | |
| CIDR_out_ Ov1^5^ | 100 | 5.9 to 21.7 | 13.1 | 0.2 | 2.7 |
| Ov1 Growth from CIDR_out_ to FTAI | 100 | -8.1 to 11.4 | 2.2 | 0.2 | 2.7 |
| **Baseline Vaginal Temperature** | | | | | |
| Baseline, °C | 131 | 37.9 to 39.3 | 38.5 | 0.02 | 0.2 |
| Baseline Duration, h | 131 | 26.2 to 57.6 | 42.7 | 0.6 | 6.4 |
| ^1^Number of observations  ^2^Age, in years, at CIDR_in_  ^2^Days post-partum on the day before CIDR_out_  ^3^Body condition and hair coat scores available at locations 1 and 2  ^4^ Fixed Time Artificial Insemination  ^5^Ov1: Presumed ovulatory follicle | | | | | |

**List of Supplemental Figures**

**Supplemental Figure 1.** Hourly ambient temperature (°C), relative humidity (%), and the temperature humidity index (THI) for years 1 (Panel A) and 2 (Panel B). Red diamonds denote time of day when a heifer first stood to be mounted by another.

**Supplemental Figure 2.** Change in heifer vaginal temperature in the 11 h before (Panel A) and after (Panel C) maximum HEAT. Impact of temperature humidity index (THI) on vaginal temperature before (Panel B) or after (Panel D) achieving maximum HEAT. Per each bin, n = the number of individual temperature measurements within each THI range.

**Supplemental Figure 3.** Hourly ambient temperature (°C), relative humidity (%), and the temperature humidity index (THI) for year 1-location 1 (Panel A), year 1-location 2 (Panel B), year 2-location 1 (Panel C), year 2-location 2 (Panel D), and year 2-location 3 (Panel E). Red diamonds denote the time of day an estrual cow reached maximum HEAT.

**Supplemental Figure 4.** Change in cow vaginal temperature in the 11 h before (Panel A) and after (Panel C) maximum HEAT. Impact of temperature humidity index (THI) on vaginal temperature before (Panel B) or after (Panel D) achieving maximum HEAT. Per each bin, n = the number of individual temperature measurements within each THI range.


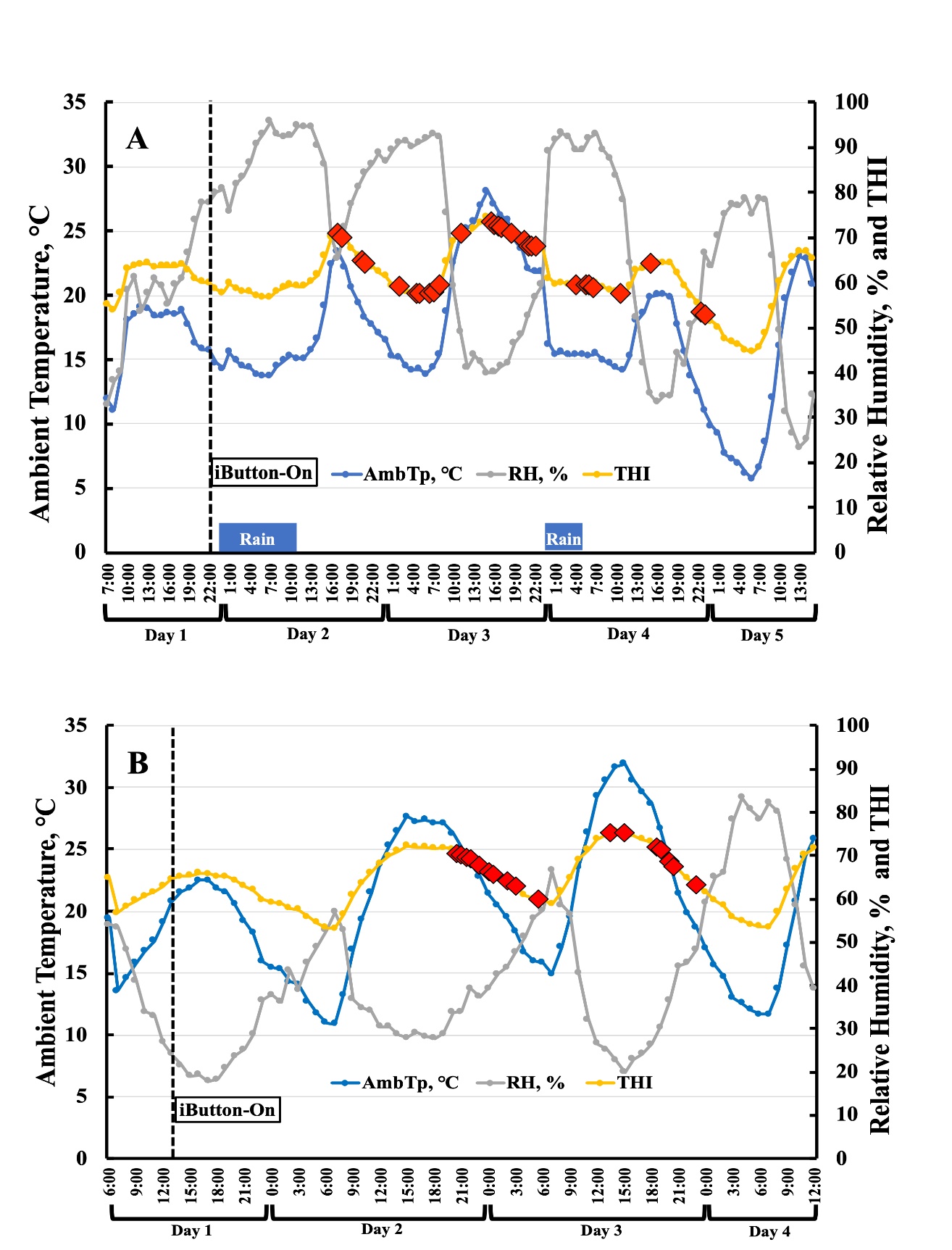


Supplemental Figure 1


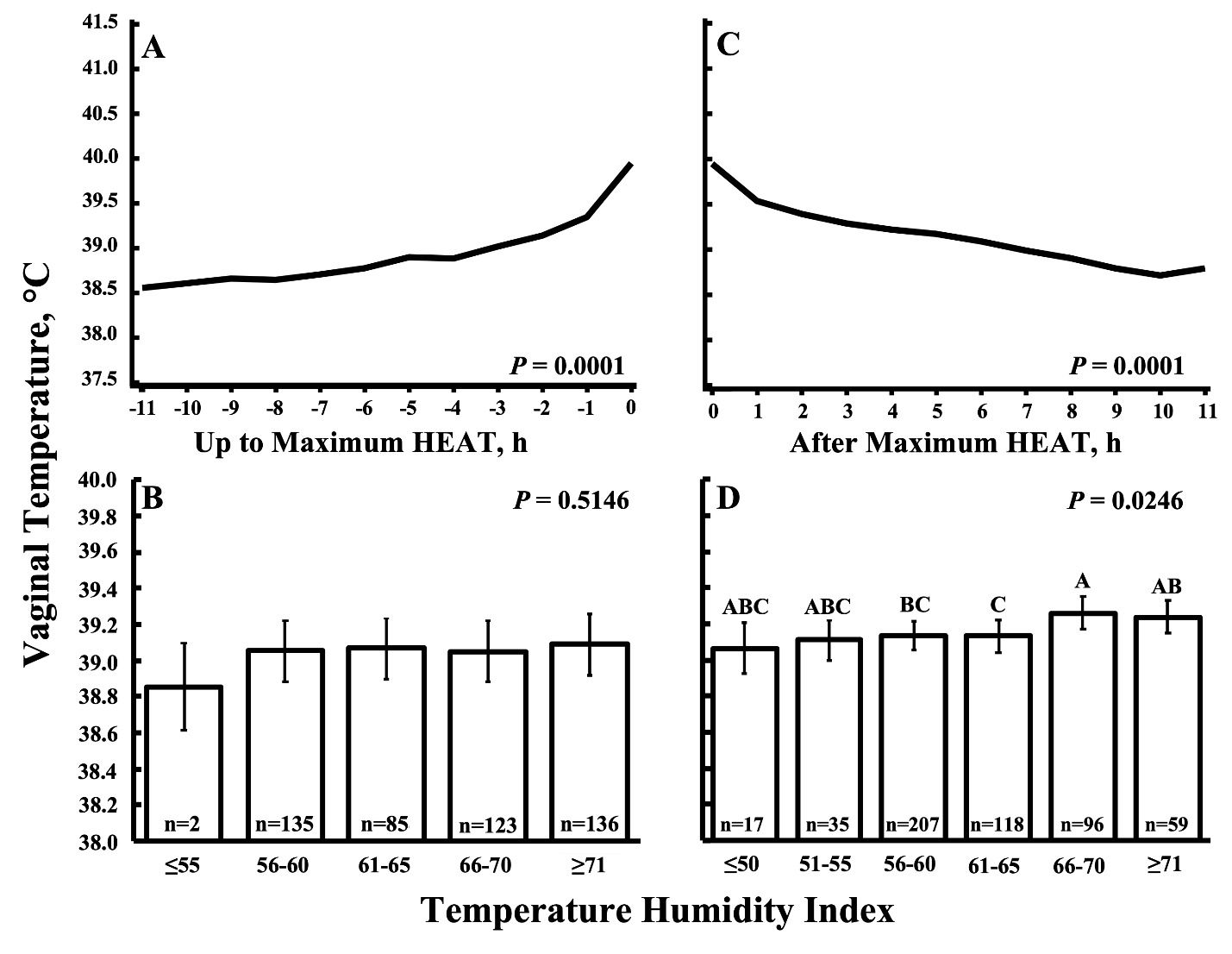


Supplemental Figure 2


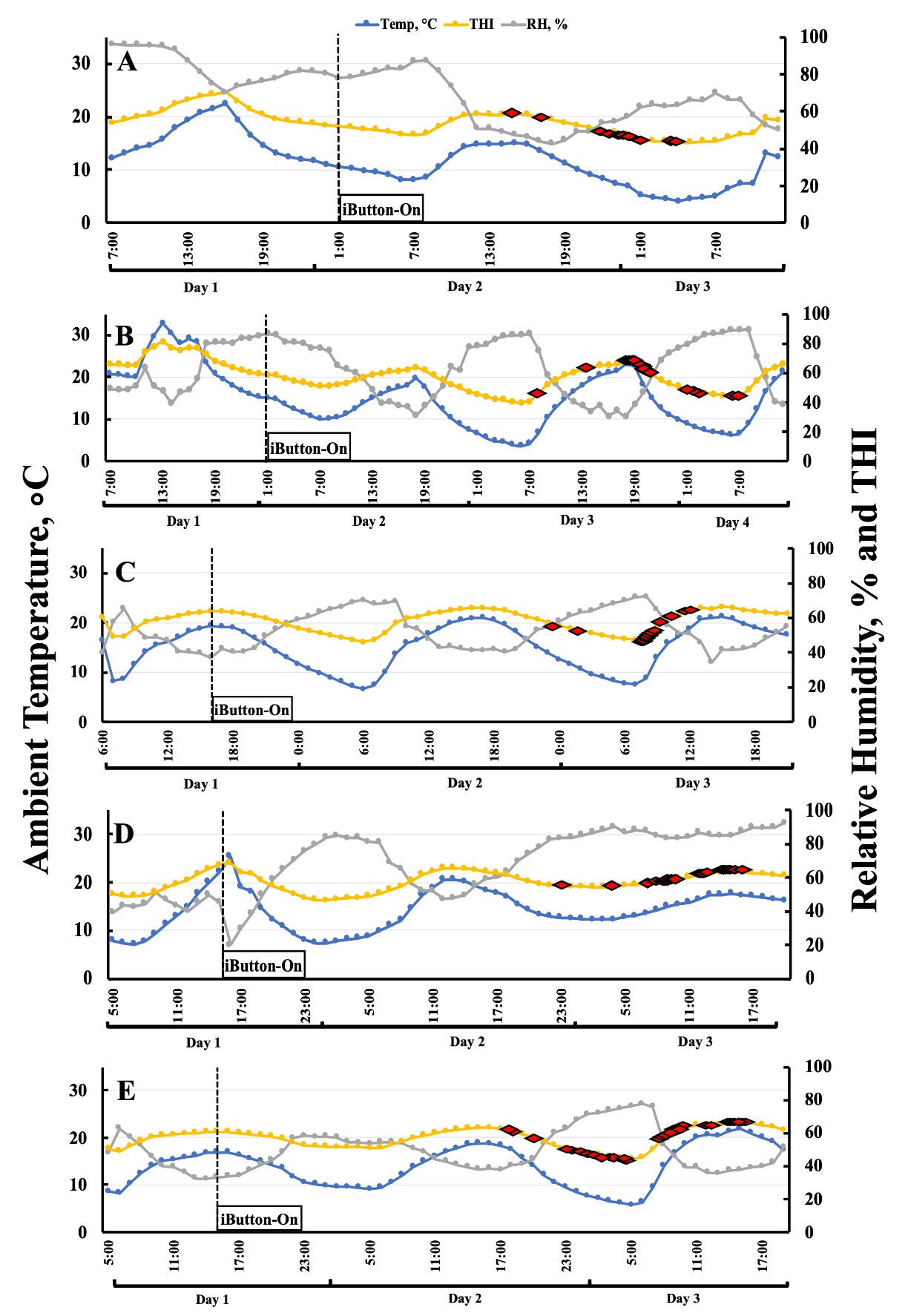


Supplemental Figure 3


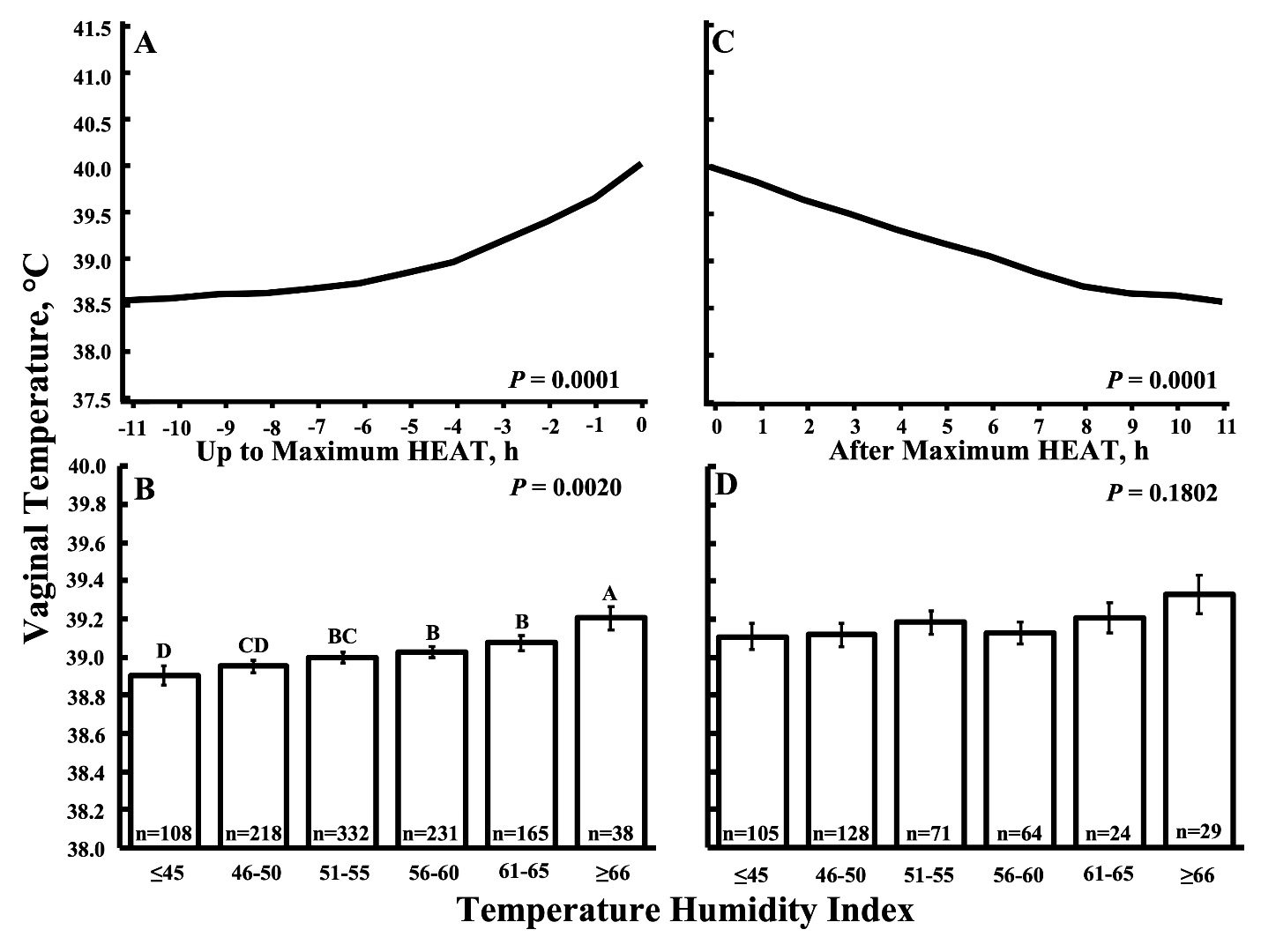


Supplemental Figure 4
